# Recent developments in catalytic materials and reactors for the catalytic pyrolysis of plastic waste into hydrogen: a critical review with a focus on the circular economy

**DOI:** 10.1039/d5ra03170b

**Published:** 2025-06-23

**Authors:** Sehar Tasleem, Abdelrahman Soliman, Edreese Housni Alsharaeh

**Affiliations:** a College of Science and General Studies, Alfaisal University PO Box 50927 Riyadh 11533 Saudi Arabia ealsharaeh@alfaisal.edu

## Abstract

Plastic waste, particularly microplastics, is a concerning environmental problem caused by the rapidly increasing production and use of plastic products as well as their improper handling. Therefore, this review presents a comprehensive critical discussion on plastic waste conversion into value-added fuels, specifically hydrogen (H_2_). This review particularly focuses on the catalytic materials employed in the catalyst-assisted pyrolysis of waste plastic into H_2_. Moreover, the advances in catalytic pyrolysis reactors are extensively discussed. Furthermore, this review considers the circular economy aspect of the pyrolysis of plastic waste in terms of the generated liquid, solid, and gas products. Lastly, the review summarizes the topic with a conclusion and future perspectives. This review offers insights into the prevailing status of plastic waste management under the circular economy framework in light of the increasing plastic waste pollution, supporting long-term sustainability.

## Abstract 2

Advancements in the catalytic pyrolysis of plastic waste to hydrogen fuel within the framework of the circular economy.

## Graphical abstract

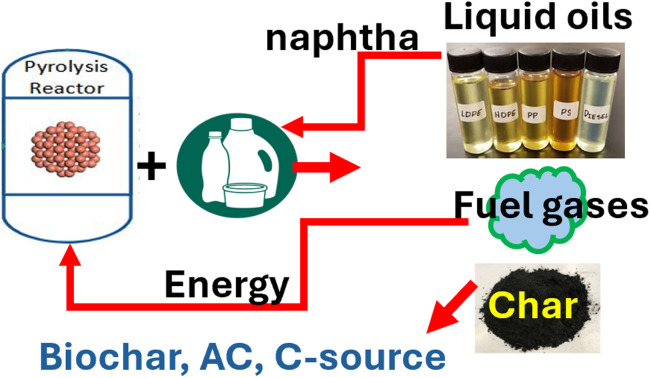

## 1 Introduction

The production of plastic is increasing globally and was estimated to reach 413.8 million metric tons in 2023,^1^ leading to significant environmental deterioration. Moreover, the world is highly dependent on the consumption of plastics owing to their high strength-to-weight ratio, strong corrosion resistance, adjustable functionality, good flexibility, and ease of processing.^2,3^ However, plastic waste, particularly microplastics, is a concerning environmental problem caused by the rapidly increasing production and use of plastic products as well as their improper handling.^4^ The majority of plastics produced are non-biodegradable and used just once before being discarded as waste.^5^ Among the most notable recent environmental crises is the buildup of this type of waste on land and in water, which is referred to as “white pollution”. As a result, managing plastic waste (PW) has become crucial. Moreover, traditional techniques such as landfilling and incineration are unsustainable and deplete resources.^6^ Therefore, as an alternative, the conversion of PW into value-added fuels, including hydrogen (H_2_), is gaining considerable attention because H_2_ energy is considered crucial to achieving the carbon neutrality target.^7^ Furthermore, the extensive use of fossil fuels has resulted in significant environmental damage; therefore, H_2_ can act as a source of clean fuel.^8^

The conversion of PW into value-added goods is a promising topic of interest for researchers. Currently, thermochemical approaches for the valorization of PW have been extensively explored, resulting from the various compositional properties of plastic to generate fuels and chemicals. The majority of H_2_ is produced commercially *via* the catalytic steam reforming of hydrocarbons to create syngas, followed by separating and purifying H_2_ from syngas.^9^ However, because of their possible financial and environmental advantages, techniques that produce H_2_ from various source materials, especially wastes and byproducts, have shown great potential.^10^ Among the approaches for managing non-biodegradable plastic waste, thermochemical treatment is a known approach that can reduce the amount of plastic waste generated and, at the same time, yield useful byproducts.^11,12^ The high content of carbon (C) and substantial calorific value of plastics make the thermochemical conversion of plastics a favorable PW management and recycling technique. Moreover, this technique is appropriate for decentralized power generation since it has the ability to recover heat and generate electricity through the use of an internal combustion engine.^13^ The thermochemical approach for plastic treatment involves combustion, pyrolysis, and gasification methods,^14^ while pyrolysis–catalysis is known to be an encouraging approach for the thermochemical conversion of plastic to valuable fuels.^15^ The main byproducts of thermochemical treatment by pyrolysis are oil and gas, which include hydrocarbon moieties. Both products can be utilized as chemical feedstocks or to produce energy.^16,17^

Numerous large-scale pyrolysis processes for PW have been developed, including batch, semi-batch, and continuous operation. The reactor range for plastic intake is 1–10 tons daily for batch reactors and 5 to 30 tons per day in continuous reactors.^18–20^ Nonetheless, there is increasing interest in using catalysts for the conversion of plastic waste into high-value products.^6^ Catalysis-based pyrolysis for the thermochemical treatment of plastic is beneficial owing to its enhanced targeted reactions and lower temperature requirements, leading to an improved overall efficiency.^15^ Moreover, the type of reactor used is also vital in PW pyrolysis, which significantly influences the mixing of PW with the catalyst, reaction rate, product yield, residence time, and product quality. Commonly, fluidized bed, fixed bed, and conical spouted bed reactors are used at the lab scale, and parameters including the type of feed, rate of feed input, pressure, temperature and mixing have a vital influence on the rate of product formation.^21^

Currently, the review articles in the literature present the conversion of plastics to value-added fuels such as H_2_, syngas, and liquid fuels, but there is a lack of comprehensive reviews specifically covering the catalyst-assisted pyrolysis of waste plastic into H_2_ with regard to catalytic materials and pyrolysis reactors. In this review, we present a broad critical discussion on the recent catalytic advancements in plastic waste conversion into H_2_ fuel *via* catalytic pyrolysis, together with an elaboration of the ideal catalyst design for plastic waste conversion to H_2_. Moreover, we also discussed the advances in reactors for catalyst-assisted pyrolysis. Furthermore, we also included the circular economy aspect of plastic waste pyrolysis in the context of the generated liquid, solid and gas products. Lastly, we summarized the conclusion and future perspectives. In this review, we specifically focus on the catalytic pyrolysis of PW to H_2_, instead of a broader discussion on PW conversion to fuels. We comprehensively discussed the catalytic materials, reactor configurations, and their roles in enhancing the H_2_ yield. In contrast to earlier studies, the use of pyrolysis products is considered, together with the circular economy. This review offers insights into the prevailing status of managing plastic waste under the circular economy framework considering the increasing PW pollution and future directions, supporting long-term sustainability.

## 2 Thermochemical conversion of plastic waste into hydrogen

The thermochemical decomposition of PW involves several processes and techniques such as pyrolysis,^22–35^ gasification,^24,36–39^ hydrothermal processes,^35,40–42^ and depolymerization^30,43–45^ for the conversion of PW into useful chemicals, fuels, and high-energy valuable materials. These processes enhance the industrial activities and environmental impacts of PW treatment globally. Each type of process has advantages for specific applications. Among them, the advantage of pyrolysis is that it facilitates the easy conversion of PW into liquid hydrocarbons and gas products, including H_2_. The operational conditions of the pyrolysis reactor control the quality and type of products generated by PW pyrolysis, including the reactor temperature, pressure, residence time, reactor design, feedstock, and selection of the catalyst. Pyrolysis can be further classified as thermal and catalytic pyrolysis.^45^ The thermal pyrolysis approach for PW conversion is conducted at extreme temperatures in the range of 300–900 °C and in the absence of oxygen to minimize the formation of char. In this process, PW is decomposed into various fractions of hydrocarbons ranging from gas fractions such as H_2_ and syngas, gasoline fractions with low contents of C_4_–C_12_ carbon, and higher viscosity liquid C_18_–C_40_ fractions. The formation of these products is typically dependent on the pyrolysis temperature and retention time, where elevated temperatures increase the formation of gas products. It is believed that the thermal pyrolysis mechanism proceeds *via* three stages/steps including initiation, propagation, and termination. In the initiation step, free radicals are produced as a result of cracking the large plastic molecules. These radicals react with the plastic molecules in the propagation step. The cracking continues, and these free radicals combine in the termination step. The products of thermal pyrolysis are wide-ranging and not selective, hence limiting their commercial value, especially given that most of these products are heavy oils. Conversely, catalytic pyrolysis is an alternative to the thermal type, which can contribute to lowering the required temperature for the decomposition process, thus reducing the total cost, increasing the selectivity of the process, directing the reaction to a specific product, and inhibiting the formation of undesirable products such as high C contents.^45^

### 2.1 Pyrolysis of plastic waste into hydrogen

Many research attempts have been conducted to produce H_2_*via* the pyrolysis of PW and its mixtures as a sustainable way to reduce their environmental impact.^35,46–48^ In these studies, the PW feedstock include polypropylene (PP),^48^ plastic mixtures, high-density polyethylene (HDPE), polystyrene (PS),^35,49^ low-density polyethylene (LDPE),^47,50^ biomass (lignin and cellulose) mixtures with PW (PE and PS),^51^ polyethylene terephthalate (PET),^52^ and cellulose biomass waste.^53^ Among them, the gaseous products from PET have the highest gas yield in the range of 52–77%, making it the most applicable plastic for gas production *via* pyrolysis. Temperature is a significant parameter in PW pyrolysis given that it controls the cracking of PW into smaller fractions. In this case, an increase in temperature will decrease the intermolecular forces among molecules and weaken the C–C bond within the molecule. To increase the yield of gaseous products, the operational temperature must exceed 500 °C.^54^ H_2_ production from PW is a favored environmental route. However, a practical difficulty, in addition to the feedstock and operating temperature, is the use of catalysts to achieve high yield at low operating temperatures.^52^

#### 2.1.1 Mechanism of catalytic pyrolysis

The mechanism of catalytic pyrolysis of plastics to H_2_ involves many interconnected chemical steps, ranging from the depolymerization of long polymer chains to gas-phase reactions. The generalized chemical equation is presented as eqn (1).1
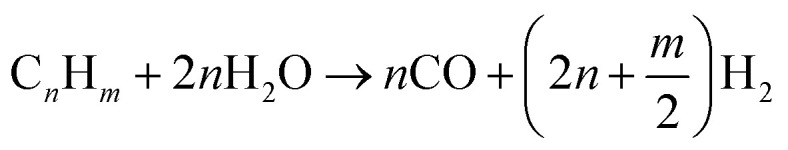
where C_*n*_H_*m*_ denotes pyrolysis-derived hydrocarbon, and 2*n*H_2_O is the steam that drives steam reforming and water–gas shift reactions.

##### 2.1.1.1 Depolymerization

**Fig. 1:**
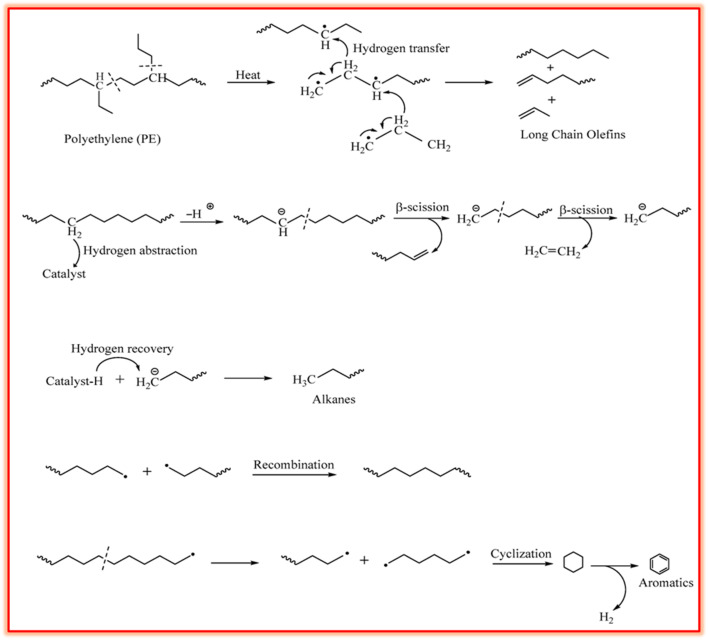
Depolymerization mechanism in PE for the formation of aromatics. Reproduced with permission from ref. 11. Copyright 2022, MDPI.

The depolymerization of plastics, ranging from initiation to product formation, as discussed in various studies, affects the composition of volatile products and their subsequent reforming, whereas the mechanism is dependent on the chemical structures of plastics.^55,56^ In the case of the commonly used PP and PE polyolefin plastics, initiation occurs *via* the creation of free radicals as a result of the cleavage of weak C–C sigma bonds,^57^ proceeding through three main mechanisms including random scission (RS), backbiting (BB), and unzipping (UZ). RS is known to be the main mechanism, involving intermolecular hydrogen transfer followed by β-scission, to produce low molecular weight compounds. In contrast, BB and UZ follow intra-chain and end-chain reactions *via* β-scission, respectively, for the generation of radicals and hydrogen atoms.^58^ PS plastics undergo depolymerization primarily through chain-end and random scission, leading to the formation of benzyl and allyl benzyl radicals, followed by β-scission for producing styrene monomers and other aromatic compounds.^59^ In polyolefins, end-chain β-scission is responsible for contributing to the C_6_–C_34_ fraction in pyrolytic oil, whereas radical recombination and hydrogen shift are responsible for the production of olefins. At elevated temperature, *i.e.*, about 400 °C, α-scission is prevalent due to the high bond dissociation energy, *i.e.*, 83–94 kcal mol^−1^, compared to that of β-scission, *i.e.*, 61.5–63 kcal mol^−1^. This leads to the generation of heavy hydrocarbons and waxes *via* α-scission and lighter gases *via* β-scission.^60^ The degradation of PE takes place *via* random chain β-scission, which leads to the formation of alkanes, alkenes, and paraffins through hydrogen transfer. Fig. 1 illustrates the underlying mechanism for the depolymerization of PE, involving free radical chain scission and hydrogen transfer, hydrogen abstraction and β-scission, and chain termination to recover proton and chain scission, followed by cyclization and dehydrogenation for the formation of aromatics.^11^

##### 2.1.1.2 Gas-phase reforming and char conversion reactions

The conversion to H_2_ occurs through a series of mechanisms involving steam reforming, water–gas shift, char formation, and char gasification.^61^ Initially, the hydrocarbons formed after pyrolysis are converted to syngas through steam reforming with the aid of steam and a suitable catalyst, as shown in eqn (2), whereas the methane steam reforming reaction is presented as eqn (3), as follows:2
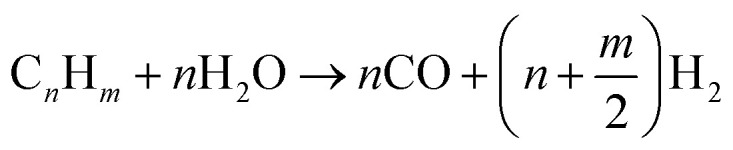

3 CH_4_ + H_2_O → CO + 3H_2_

The CO generated from steam reforming undergoes the water gas shift reaction, increasing the yield of H_2_, which is considered a major process in pyrolysis–reforming (eqn (4)).

4 CO + H_2_O → CO_2_ + H_2_

Not all the pyrolytic intermediates are transformed into volatiles, thus forming C-rich char consisting of C, ash, and some trace metals. This C-rich char undergoes gasification, which converts it into syngas, increasing the yield of H_2_, and at the same time, eliminating solid residue. eqn (5) and (6) show the primary gasification step, which is the water–gas shift reaction, where solid C reacts with steam to generate syngas, and the secondary water–gas shift reaction, which takes place together the primary step, where excess steam reacts with C to generate more H_2_, and also CO_2_. Eqn (7) presented the Boudouard reaction, where the generated CO_2_ reacts with C.

5 C + H_2_O ↔ CO + H_2_

6 C + 2H_2_O ↔ CO_2_ + 2H_2_

7 C + CO_2_ ↔ 2CO

## 3 Catalytic advances in plastic waste conversion to hydrogen

**Fig. 2:**
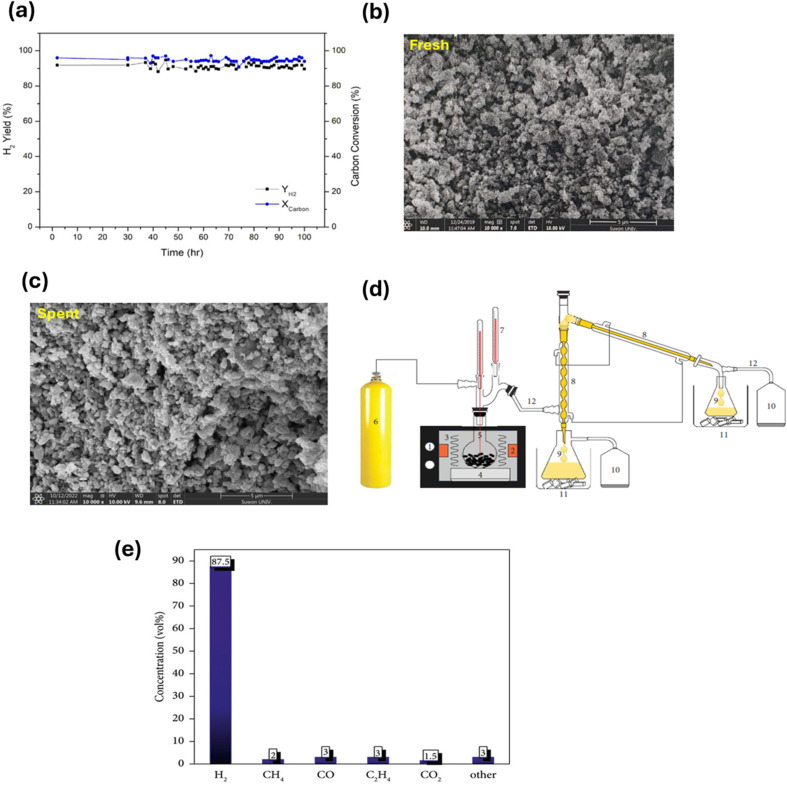
(a) Durability test of the Ni/Ce–Zr–Mg/Al_2_O_3_ catalyst. SEM images of Ni/Ce–Zr–Mg/Al_2_O_3_: (b) fresh sample and (c) spent sample. Reproduced with permission from ref. 75. Copyright 2023, MDPI. (d) Setup used for the pyrolysis of HDPE, consisting of (1) a microwave source, (2) waveguide, (3) microwave interaction with feedstock, (4) ceramic platform, (5) feedstock container, (6) nitrogen supply, (7) temperature sensor, (8) condenser, (9) oil container, (10) gas sampler, (11) cold trap, and (12) glass tubing. (e) Gas production over NiZnFe_2_O_4_, NiMgFe_2_O_4_ and MgZnFe_2_O_4_ in the microwave-assisted catalytic pyrolysis of plastic waste. Reproduced with permission from ref. 83. Copyright 2024, Wiley.

Generally, catalytic pyrolysis is superior to thermal pyrolysis due to many factors, including its lower energy demand given that it operates at lower temperatures with faster rates, shortening the reaction time, and enables better control and higher quality product formation by directing the reaction to produce specific products.^62^ Catalysts can be classified as homogeneous, which are less common, or the most common heterogeneous catalysts. The popular heterogeneous catalysts include zeolites,^51,63–65^ nickel-based catalysts,^51,66^ silica-alumina, transition metals, and their oxides or metal alloys.^67,68^ Catalyst design significantly impacts the efficiency and yield of the processes for the pyrolysis of plastic waste. It influences the product quality, optimizes the reaction conditions, and enhances the recovery of valuable materials.^69^ The catalyst design aims to enhance the sustainability of the waste management process and resist deactivation caused by coking by implementing different composites as alloy, oxide, or mixed oxide catalysts. In this section, we discuss the different types of catalysts employed in the pyrolysis of plastic waste. Table 2 summarizes the various catalysts employed for the pyrolysis of PW into H_2_, together with the feedstock, reaction conditions, and selectivity. The nickel-based catalysts are some of the most commonly used catalysts for the production of H_2_ from PW through the pyrolysis–catalytic steam reforming process, and they are even preferred over other transition metals, such as Cu, Co, and Fe, and the noble metals, such as Pt, Ru, Rh, and Pd. Specifically, nickel catalysts are selected over other catalysts because of their high activity in breaking C–C, C–H, C–O, and O–H bonds, their low cost, and high affinity for the generation of H_2_.^70,71^ Nickel-based composites are also utilized for the conversion of biowaste and PW into H_2_. Atong *et al.*^66^ utilized Ni/SiC composites as catalysts for the conversion of waste glycerol *via* pyrolysis into syngas and methane. The operating temperature was set above 600 °C to obtain higher conversion efficiency. The complete conversion of glycerol into fuel gases was achieved at 800 °C *via* pyrolysis gasification processes. Another study utilized NiO/La_2_O_3_ for the production of H_2_ by steam-reforming ethanol. This catalyst showed high catalytic activity at low temperatures and had high catalytic stability for more than 1000 h after 13 cycles.^72^ Wang *et al.* introduced porous CaO to support Ni/Al_2_O_3_ catalysts for *in situ* CO_2_ capture for H_2_ production from biomass gasification. NiO particles were distributed over the porous CaO. This catalyst showed great resistance to carbon deposition on NiO/CaO, resulting in lower coke deposition and higher H_2_ production compared with Ni/Al_2_O_3_ catalysts.^73^ Zhang *et al.*^74^ developed the Ni–La/Al_2_O_3_–CeO_2_ catalyst for the pyrolysis of biomass and polyethylene to H_2_. The introduction of La improved the catalytic performance and stability. Han *et al.*^75^ produced H_2_*via* PW pyrolysis over the Ni/Ce–Zr–Mg/Al_2_O_3_ catalyst using steam reforming. This catalyst showed high stability for 100 h (Fig. 2(a)), which was also evident from the SEM images, as shown in Fig. 2(b and c), and the H_2_ yield was 91.3%. Another work developed a ternary NiMo–Bi liquid alloy catalyst for the CO_2_-free pyrolysis of methane into H_2_ at moderate temperatures. The catalyst operated between 450–800 °C, and at higher temperatures, it was 100% selective for H_2_ production with 120 h of stability. Normally, nickel-based catalysts suffer from deactivation due to the formation of carbonaceous residues.^76^ Haryanto *et al.*^77^ utilized a ceria Ni-supported catalyst on alumina (Ni/CeO_2_–Al_2_O_3_), which showed the best performance in the production of H_2_*via* the water–gas shift process. The addition of small amounts of cobalt or chromium resulted in an increase in the catalytic performance of the catalysts employed in this study. The operational temperature was 450 °C, and the catalytic performance was compared with a commercial catalyst (Shift Max 120). In the current work, a two-stage reactor was used to study the evolution of H_2_*via* the pyrolysis–catalytic steam reforming of PET, PS, and ethylene. With 10 wt% Ni/AlO_3_, PS produced the highest H_2_ yield (125 mmol g_plastic_^−1^) at 900 °C and a steam input weight hourly space velocity of 7.59 g h^−1^ g_catalyst_^−1^. The H_2_ production was greatly increased by a high catalyst temperature and an ideal steam input.^78^ In a recent work, Sathish *et al.* used pyrolysis to turn PW, such as milk pouches and bottle wrappers, into carbon and H_2_ nanoparticles. The procedure was performed using different catalysts (Ni/SiO_2_, Co/SiO_2_, and Ni/Mg), temperatures (400–500 °C), and reaction durations (30–50 min). Consequently, improved gas yield and H_2_ conversion were achieved using a longer reaction period (50 min) and higher temperature (500 °C). Also, Ni/SiO_2_ demonstrated the best performance with an H_2_ conversion efficiency of 12.8% and production of 34.7 g of carbon nanoparticles.^79^ Song *et al.* improved low-temperature H_2_ generation from PE by utilizing an NiCeO_*x*_/β catalyst with nonthermal plasma. NiO and CeO_2_, as metallic and acidic sites, respectively, were found to be important active centers. A high H_2_ yield of 32.71 mmol g^−1^ and 82.1% selectivity were attained at 400 °C, 210 W, and PE/catalyst ratio of 1/4. Effective PE pyrolysis at low temperatures was made possible by nonthermal plasma, which promoted effective plasma–catalyst interactions.^80^

Iron-based catalysts have also been employed in H_2_ production *via* different feedstocks and varying operational conditions. The main advantage of iron catalysts is that they are more stable at high temperatures and more resistant to carbon deactivation compared with nickel catalysts. Another work reported the use of an Fe_2_O_3_/Al_2_O_3_ catalyst for the production of H_2_*via* the catalytic–pyrolysis of high-density and low-density PE. The synergetic catalytic activity of both iron and aluminum oxides was superior to that of the individual oxides.^81^ A study utilized 10% Fe_2_O_3_/90% CeO_2_ and red mud to produce H_2_ from algal biomass, where the catalyst mainly converted tar produced form algae pyrolysis into H_2_. The steam-gasification experiments were conducted at temperatures in the range of 600–850 °C. The reduction of tar levels was in the range of 80–100% for seaweeds and 53–70% for microalgae, indicating the effect of components of biomass on the conversion rate of tar into H_2_.^82^ In a recent study, Shoukat *et al.* utilized magnetic ferrite catalysts, *i.e.*, NiZnFe_2_O_4_, NiMgFe_2_O_4_ and MgZnFe_2_O_4_, for the microwave-assisted catalytic pyrolysis of PW into nanostructured carbon and H_2_ fuel, as shown in Fig. 2(d). Among the magnetic catalysts, the NiMgFe_2_O_4_ catalyst showed the best performance in H_2_ production (Fig. 2(e)).^83^ Another study conducted the plasma–catalytic pyrolysis of polypropylene over Fe/γ-Al_2_O_3_ in a dielectric barrier plasma discharge reactor for the production of H_2_ and carbon nanotubes (CNTs). The plasma reactor decreased the temperature for the formation of CNTs by 100 °C due to the cleavage of the volatile products of pyrolysis, promoting the conversion of liquid and gaseous products to CNTs and H_2_.^26^ Biomass mixed with plastics including high-density PE, PP, and PS was used in pyrolysis/gasification over Ni-based catalysts, where significant H_2_ and CO_2_ evolved in the gaseous fraction as a result of the outstanding performance of the Ni/Al_2_O_3_ catalyst in the gasification process, which appears to effectively encourage water–gas shift and steam reforming reactions.^35^ In another work, an alumina support having a 10 wt% loading of iron was used to convert light-density PE into CNTs and H_2_ using a two-stage pyrolysis–catalytic reactor. The yield and quality of CNTs were improved at higher temperatures, while uniform CNTs were produced at 800 °C. Because of the increased carbon transport, both CNT creation and H_2_ generation increased with an increase in temperature. The increase in plastic input enhanced the CNT yield up to a certain point, but an excessive loading (*e.g.*, 1.25 g) decreased the efficiency and increased the production of amorphous carbon.^47^ In a recent study, FeO_3_/AlO_3_ was used for the catalytic pyrolysis of high-density PE and demonstrated improved chain cracking and C–C/C–H bond cleavage compared to pure catalysts. It produced 50.53 mmol g^−1^ of H_2_, which is more than 70% of the H_2_ content of the plastic and hydrocarbon products ranging from C_2_ to C_9_. Proton adsorption and C–H bond cleavage were improved by the production of FeAlO_4_ during pyrolysis and catalyst–support interactions, which were linked to the improved performance.^84^ Plastics were quickly broken down into H_2_ and high-value multiwalled CNTs in as little as 30 to 90 s *via* the microwave-assisted catalytic deconstruction of different plastic feedstocks utilizing low-cost iron-based catalysts. By extracting more than 97% of the theoretical H_2_ content of the plastic in a single step, this approach produced a high H_2_ yield of 55.6 mmol, proving to be a scalable and effective way to value plastic waste.^49^ Using SN5-800 12 nickel-modified sepiolite, mixed PW containing rigid PP, expanded PS, high-impact PS, and PE was pyrolyzed in two stages, yielding up to 27.2 mmol H_2_ per g at 800 °C. Treating sepiolite with acid increased the H_2_ yield to 26.4 mmol g^−1^. Only about 20% of the carbon deposits was filamentous; the majority were amorphous.^85^ The catalytic pyrolysis of PE was studied using Fe/ZSM-5 catalysts with different Fe loadings (5–30 wt%). At an Fe loading of 20%, 262.24 mg g_PE_^−1^ of CNTs and 31.72 mmol g_PE_^−1^ of H_2_ were produced. Although more active sites were created by a higher Fe content, an excessive loading (30%) resulted in particle agglomeration, which inhibited CNT development and promoted the formation of carbon nanofibers and nano-onions.^86^

Activated and carbonaceous catalysts have also been employed in pyrolysis processes for H_2_ production. Zhang *et al.*^87^ fabricated catalytic carbon membranes by mixing phenolic resin and a nanocopper-based catalyst. The prepared catalyst was utilized in H_2_ production *via* methanol steam reforming. The results indicated that the prepared catalyst remained stable for a long time and the of conversion was efficient, achieving the H_2_ of 92%. Another study utilized Ni-activated carbon (Ni-AC), Fe-activated carbon (Fe-AC), and Zn-activated carbon (Zn-AC) to produce H_2_ and CNTs *via* PW pyrolysis in a two-stage fixed bed reactor. The temperature in the 1st stage was 500 °C, and in the second stage, it was in the range of 500–700 °C. The production of H_2_ was superior to that of the Ni-AC catalyst under the operating conditions. These results were compared with the commercial zeolite catalyst H-ZSM-5.^23^ An Fe-based catalyst on AC for high-yield H_2_ generation from PP was explored. The water content, catalyst quantity, and Fe loading were shown to be important factors in this process. When the optimum content of water was added, the H_2_ yield increased from 38.73 mmol g_PP_^−1^ without water to 112.71 mmol g_PP_^−1^. Although the H_2_ selectivity declined over time, the 15 wt% Fe/AC catalyst demonstrated good stability over ten cycles.^88^ To improve the pore structure and active sites of AC catalysts, co-doping metals, including Fe, Co, Ni, and Zn, with nitrogen, was explored. The 10%Fe/N-AC catalyst outperformed 10%Fe/AC in terms of surface area and microporosity. The pyrolysis of corn stover with high-density PE using 10%Fe/N-AC led to the generation of 60.3% monoaromatic hydrocarbons and 19.5% polycyclic aromatic hydrocarbons. Alternatively, 10%Ni/N-AC generated 56.2% H_2_.^89^

**Fig. 3:**
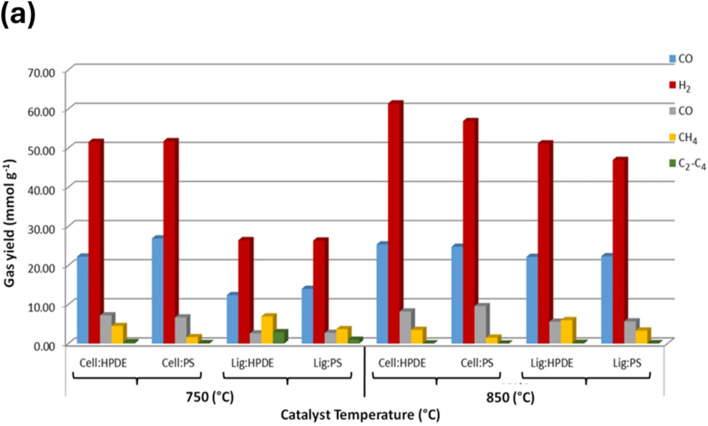
(a) Gas composition output from the co-pyrolysis steam reforming of biomass components with PW, demonstrating the effect of temperature on the process. Reproduced with permission from ref. 51. Copyright 2022, Springer.

Low-cost catalysts such as zeolites, clays, and bimetallic materials have been employed in the catalytic-assisted pyrolysis of PW and biomass waste to decrease the cost and pyrolysis temperature of the process.^90^ In a study on H_2_ production from PW, H_2_ was produced from PP waste using a two-stage screw kiln, which involved catalytic gasification after pyrolysis. Higher gasification temperatures (600–900 °C) and better water injection greatly enhanced the H_2_ production using the Ni–Mg–Al catalyst. The highest H_2_ yield of 22.38 g per 100 g of PP (52% of the theoretical maximum) was obtained at 800 °C with a water injection rate of 28.46 g h^−1^.^46^ In the study by Akubo *et al.*, cellulose and lignin were co-pyrolyzed with PW, including PE and PS, in the pyrolysis–catalytic steam reforming process to produce H_2_. The catalyst used was 10% Ni/MCM-41. The catalyst was compared with Ni/Al_2_O_3_ and Ni/Y-zeolite supported catalysts. Cellulose/plastic mixtures produced a higher H_2_ yield compared with lignin/plastic. Upon increasing the catalytic steam reforming temperature from 750 °C to 850 °C, the opposite effect was evident, as shown in Fig. 3(b). The best catalyst to produce H_2_ and syngas was found to be Ni/Al_2_O_3_ compared with other catalysts.^51^ However, a drawback of catalytic pyrolysis is the deactivation of the catalyst due to the formation of coke. The goal is to utilize stable catalysts with easy regeneration; thus, utilizing zeolites in the pyrolysis of plastic waste can be a potential solution.^91^

Microwave radiation facilitates the pyrolysis process and has many advantages over conventional heating *via* rapid heating, which reduce the production costs. Samples are directly heated by microwave irradiation; this direct interaction shortens the heating time. Thus, many studies have applied the microwave-assisted pyrolysis for the production of H_2_ from PW (Table 1).^49,53,92–94^

**Table 1:** Summary of various catalysts employed for the catalytic pyrolysis of PW into H_2_, together with the feedstock used, reaction conditions, and selectivity

Catalyst	Feedstock	Reaction conditions	Conversion	Selectivity	Remarks	Reference
Ni–Mg–Al	PP	600–900 °C	PP to H_2_	52% of the optimum theoretically H_2_ available in the PP was achieved	The gasification temperature was 800 °C, and the rate of water injection was 28.46 g h^−1^	48
Ni/Al_2_O_3_	Biomass/PP/PE/PS	600–800 °C	Water–gas shift of producing H_2_ from biomass/PW mixtures	56.9 wt% of H_2_ yield	H_2_ and CO_2_ evolved in the gaseous fraction	35
Iron catalyst	Light-density PE	700–900 °C	Conversion of light-density PE into CNTs and H_2_	—	10 wt% loading of iron was optimum, and uniform CNTs were best produced at 800 °C	47
Cu/ZnO/Al_2_O_3_ catalytic carbon membranes	Methanol	25–24 °C	Methanol to H_2_	92% for H_2_ output and 95% for methanol conversion was achieved	CCM-assembled reactor 9.6 h^−1^ space velocity and methanol/steam ratio 1 : 1	87
Ni/MCM-41	Lignin and cellulose/PE and PS mixture	750 to 850 °C	Biomass/biopolymer to H_2_	The production of syngas, as well as H_2,_ was enhanced at a steam/feed ratio of 3 : 85	A fixed-bed reactor was used	51
Ni/Al_2_O_3_						
La/Al_2_O_3_–CeO_2_-bamboo charcoal	PE	700 °C	Straw/PE to H_2_	H_2_ yield of 332.2 mL g^−1^	The optimum straw/PE ratio was 5 : 5, and the catalyst was highly stable	74
Ni/Ce–Zr–Mg/Al_2_O_3_	Naphtha oil	750–850 °C	Steam reforming process of oil to H_2_	Naphtha oil (C_6_–C_7_) produced H_2_ from catalytic pyrolysis of PW	H_2_ yield of 91.3% was obtained	75
Fe_2_O_3_/Al_2_O_3_	High-density PE	800 °C	Pyrolysis–catalytic decomposition of plastics	The H_2_ yield with Fe_2_O_3_/Al_2_O_3_ was more than 70% of hydrogen in plastic	Pyrolysis–catalytic decomposition of plastics	84
Ni–Mn–Al	PP	800 °C	PP to H_2_ and CNTs	H_2_ production along with CNTs	Pyrolysis catalytic reforming was employed	46
FeAlO_x_	High-density PE, PP, PS	550 °C	PW into H_2_	H_2_ levels were about nearly 90 vol% and yield of H_2_ was 55.6 mmol g_plastic_	A quick and easy one-step method for breaking down PW with microwaves	49
Ni/Al_2_O_3_	PE, PS, and PET PW	700 °C to 900 °C	Plastic mix to H_2_	H_2_ yield was 125 mmol g_plastic_^−1^	Pyrolysis catalytic steam reforming was employed	78
Fe_2_O_3_/Al_2_O_3_	High-density PE	800 °C	PE to H_2_	H_2_ yield of 50.53 mmol g_plastic_^−1^ was obtained. It amounted to about 70% of the H_2_ in plastic	—	95
Ni/SiO_2_	Milk pouches and bottle wrappers	500 °C for 50 min	Plastic to H_2_ and carbon nanoparticles	—	H_2_ conversion efficiency of 12.8% and the production of 34.7 g of carbon nanoparticles	79
SN5-800 12 nickel-modified sepiolite	Rigid PP, expanded PS, high-impact PS, and PE	800 °C	PW mix to H_2_ and carbon	H_2_ production with only about 20% of the filamentous carbon deposits; the majority were amorphous	Sepiolite treated with acid increased the H_2_ yield to 26.4 mmol g^−1^	85
NiCeO_*x*_/β	PE	400 °C	PW to H_2_	H_2_ production	The conversion process was low-temperature nonthermal plasma-assisted catalytic pyrolysis	80
Fe/ZSM-5	PE	800 °C	PE to H_2_ and CNTs	CNTs and H_2_	20 wt% Fe produced maximum H_2_ and CNTs	86
Fe/AC	PP	PP to catalyst ratio 1 : 0.75, water content 6 mL h^−1^	PP to H_2_	H_2_ production selectivity decreased with increasing temperature	Adding water to the reaction was beneficial for improved H_2_ evolution	88
Fe, Co, Ni, and Zn, co-doped nitrogen AC	Corn stover + high-density PE	—	Plastic + biomass to H_2_ and hydrocarbons	Monoaromatic hydrocarbons, polycyclic aromatic hydrocarbons, H_2_	Co-doping AC with nitrogen and metals (such as Fe and Ni) greatly enhances the surface area, porosity, and active site density of the catalyst	89

## 4 Advances in reactor design for the catalytic pyrolysis of plastic waste into hydrogen

Many types of reactors, having varying configurations, have been developed and employed for plastic PW to H_2_ evolution through a 2-stage pyrolysis catalytic steam reforming process. Table 2 summarizes the various types of reactors employed for the catalytic pyrolysis of PW to H_2_, together with their compatible plastic types, operation mechanisms, advantages, and disadvantages. In stage 1 of the process, a lower temperature of approximately 500 °C is used to carry out the pyrolysis of PW, generating a mixture of vapors and hydrocarbon gases. In stage 2, the process of reforming takes place where the generated gases pass through the catalytic reactor at approximately 800 °C with steam and a catalyst to produce gas containing H_2_.^96^ Next, the produced gas is passed through a condenser to cool it for the removal of condensable hydrocarbons.^15^ Several complex experimental configurations have been established for pyrolysis–catalytic reforming, including screw kiln fixed bed, spouted bed pyrolysis, pyrolysis fluidized bed reforming continuous system, and pyrolysis-fixed bed reforming. Various reactor designs are advantageous for optimizing the process parameters, including temperature, catalyst, and type of PW to get a greater H_2_ yield.^97^

**Table 2:** Summary of various types of reactors employed for catalytic pyrolysis of PW into H_2_, along with their compatible plastic type, operation mechanisms, advantages, and disadvantages

Reactor type	Type of plastic feed	Operation mechanism	Advantages	Disadvantages
Fixed bed	Low- and high-density PE and PP^140–142^	The catalyst is pelletized and kept stationary^143^	Less costly setup and operations, longer residence time to more conversion rates of C, more char production, and less ash carryover^144,145^	Up-scaling challenges, less heat and mass transfer, difficult tar elimination, and poor production of gaseous and liquid products^146,147^
Fluidized bed	Low density PE, PP, PE^148–150^	Fluidization helps to combine PW with the catalyst and allows continuous operation^143^	Adaptable reaction procedures, good heat and mass transfer, high temperature working, controllable vapour holding time, and can be easily scaled up for the generation of bio-char^143^	Expensive pre-treatment, challenging to separate the catalyst bed from the char, challenging to work with small-sized particles of material, the feeding setup gets blocked easily, corrosion issue, and it is difficult to carry defluidization upon melted plastic sticking to the material bed.^54,115^
Screw kiln	Low-density PE, PS, and PP^151^	A screw or auger rotates within a tube or cylinder^152^	Heating is uniform, and different types of feeds can be processed^153^	Less heat transfer, difficulty controlling temperature, low scalability, more costly, friction problem due to the interaction of the walls of the kiln drum and materials^153^
Conical spouted bed	Low- and high-density PE, PP, PS, PET, PE, and poly(methyl methacrylate)^108,154,155^	An effective flash pyrolysis and constant feed substitute for fluidized bed reactors^143^	Handling of different particle shapes, sizes, and densities, less rate of attrition, and lower segregation of the catalyst bed^156–158^	Catalyst feeding, difficulty in collecting products, *i.e.*, liquids and solids
Rotary kiln	PE, PP, PS, and a mixture of PW^159^	Rotating drum or set of blades moves the material^152^	Materials are heated and mixed well, even production of products, residence time can be adjusted, low maintenance, various particle-sized materials can be fed^160^	Heating is slow, and more char production^160^
Microwave assisted	PP, PS, PP + PS, low- and high-density PE, PET, PVC, and halogenated PW^124^	Uses microwaves to promote energy transfer through atomic or molecular contact^124^	Efficient heat transfer, highly efficient, higher rate of heating, low energy input, good recovery of chemicals, efficient gas and oil product generation^143,161^	It requires more capital, requires microwave absorbers, measuring temperature is an issue, less efficient mixing, and the challenge of controlling the size of PW particles^161^
Plasma assisted	Low and high density PE, PP, mix PW^134^	PW is kept in a cylindrical tube, installed with two Cu electrodes^143^	Faster rate of reaction, less production of tar, good reaction kinetics, and higher energy density^160,162^	Greater cost in terms of operation, and it is energy-intensive^163^

### 4.1 Fixed and fluidized bed reactors

**Fig. 4:**
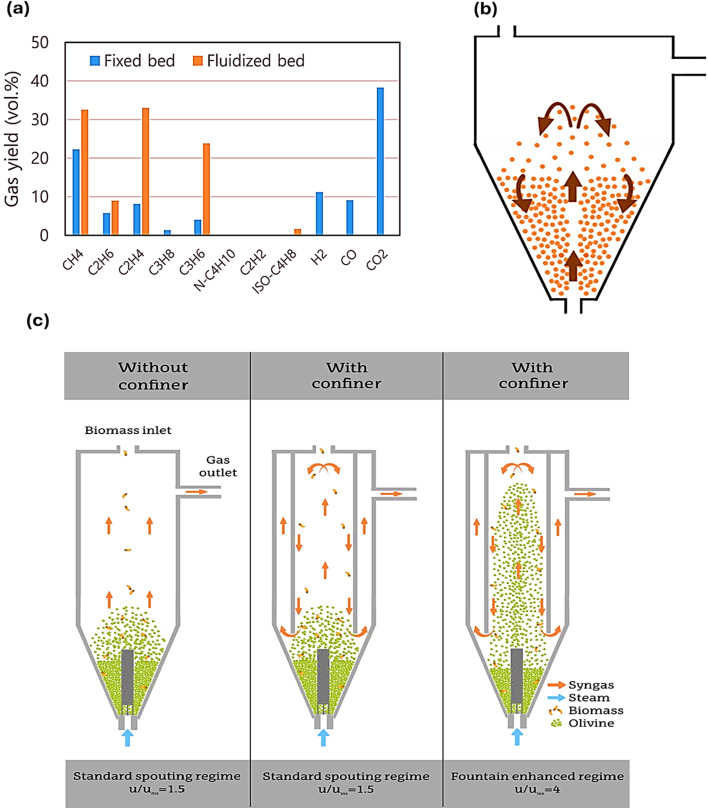
(a) Volume% yield of gases generated *via* the pyrolysis of acrylonitrile butadiene styrene plastic using a fixed bed reactor and fluidized bed reactor, analyzed using GC. Reproduced with permission from ref. 101. Copyright 2024, Elsevier. (b) Illustration of a typical conical spouted bed reactor for plastic pyrolysis. Reproduced with permission from ref. 108. Copyright 2025, Royal Society of Chemistry. (c) Various spouting regimes and patterns for flow of gas in conical spouted beds without a confiner, with a confiner, and with a confiner in the fountain enhanced regime. Reproduced with permission from ref. 110 Copyright 2025, Elsevier.

In terms of a fixed bed reactor, recently, a three-stage reactor was utilized to produce H_2_ from waste polypropylene involving (1) pyrolysis, (2) steam reforming in the presence of a catalyst, and (3) water–gas shift in 3 different reactors within the same configuration. A temperature-controlled, electronically heated furnace was provided for external heating in the first stage, which was conducted in a stainless-steel reactor. A stainless-steel container, secured within the center of the pyrolysis reactor, was filled with 1 g of PP, which was heated from 20–500 °C for a duration of 20 min. Next, catalytic steam reforming occurred when the generated hydrocarbons were passed to a reforming reactor maintained at 850 °C and containing Ni/Al_2_O_3_ as the catalyst. Lastly, the product gases generated in the reforming reactor were transferred to a hot water gas shift reactor, where, with the help of a metal–alumina catalyst, the gases containing H_2_ and CO underwent a catalytic water–gas shift process. Consequently, 122 mmol g_plastic_^−1^ was achieved using 5 wt% Fe/Al_2_O_3_ catalyst. This study also demonstrated the disadvantages of using a fixed-bed catalytic pyrolysis reactor in terms of H_2_ yield. Higher H_2_ yields of 168 g_plastic_^−1^ and 185 g_plastic_^−1^ were reported for the pyrolysis of hydrocarbons in a catalytic steam reforming reactor with a fluidized bed. The high production of CO during the water–gas shift reaction would be achieved as the catalytic steam reforming in the reforming reactor increases, leading to maximized H_2_ production. Furthermore, the water–gas shift reactor only operates at one temperature, while independent reactors working at high temperature and low temperature with temperature controllers allow an enhanced H_2_ output.^98^ The fluidized bed reactor and spouted bed reactors possess greater efficiency in terms of mass and heat transport than the fixed bed reactor.^99^ Furthermore, given that it has the capacity to efficiently mix feedstock and attain extreme heating, it is typically used to investigate the behavior of fast pyrolysis. One major benefit of fluidized bed reactors is their direct flexibility in achieving the required product distribution through control of the operational parameters such as temperature. Moreover, owing to the presence of a heated fluidizing medium inside the reactor, fluidized bed reactors exhibit superior heat transfer and mass transfer capabilities.^100^ The performance of fixed bed and fluidized bed reactors for the pyrolysis of plastic waste was compared in a recent study, where 5 kg of sand was used to form the sand bed in the fluidized bed reactor. The ideal temperature for pyrolysis was 520 °C, given that it provided the optimum operational stability, and the flow rate of 12 L per min N_2_ was chosen for the fluidized bed reactor, whereas 5 mL min^−1^ for the fixed bed reactor. The fluidized bed reactor processed 264.6–284.4 g of PW in 30 min, whereas the fixed bed reactor processed about 280 g of PW over 30 min at the same feeding rate. According to the results, the fluidized-bed reactor led to a 26–38% increase in the generation of light chemicals, *i.e.*, C_5_–C_10_, compared to the fixed bed reactor, as well as an 8.6–38.1% increase in the fluidized-production of C_1_–C_2_ gases including CH_4_ and C_2_H_4_, which was the result of C–C bond scission reactions. Alternatively, more C_3+_ gases were produced in the fixed bed reactor in comparison to the fluidized bed reactor, which can be ascribed to its exceptional heat transfer efficiency, which increased the exact pyrolysis temperature. However, no H_2_, CO_2_, and CO was generated by the fluidized bed reactor, as shown in Fig. 4(a). This can be attributed to the fact that the high flow rate in the fluidized bed reactor purged the reactor completely of air compared to the fixed bed reactor, where some air was left.^101^ The existence of essential parameters that impact the gasification process is another significant problem when gasifying PW. In other words, the quality and quantity of the product gas linked to the chosen feedstock can be greatly impacted by the operating parameters such as temperature and pressure, type of reactor, and type of gasifying agents.^102^ In a recent study, Aspen Plus® was used to create a thermodynamic equilibrium model to simulate the generation of H_2_*via* the air gasification of five distinct types of plastics. Experimental data were used to validate the numerical model. Parametric studies were performed to examine the impact of variables such as gasification temperature, pressure, and equivalence ratio (ER) on the syngas composition, syngas lower heating value (LHV), H_2_ production, and lower heating value. The study found that (1) the gasification temperature has a significant impact on the syngas composition. Increasing the temperature improves the system performance, which raises the H_2_ yield. The most important process that influences the composition of gas produced during gasification is the water–gas shift reaction. At high temperatures, the water–gas shift reaction, which produces H_2_, is likewise unfavorable. The water–gas shift reaction and steam methane reforming reaction contribute to increasing the H_2_ concentration prior to 750 °C. After that, the reactions are limited by the shortage of reactants such as CH_4_ and steam, which reduces the H_2_ concentration. (2) A fluctuation in ER has a greater impact on the system performance than the gasification temperature. A higher ER results in a lower syngas LHV and H_2_ generation, which is attributed to the fact that boosting the level of O_2_ in the system by adding more gasifying agent results in a higher ER. The gas components are greatly impacted by an increase in O_2_ content. Increased O_2_ concentrations encourage H_2_ and carbon oxidation processes, which increase the concentration of CO_2_ and H_2_O. As a result, as the ER increases, the H_2_ concentration decreases. (3) As the gasifier pressure increases, the concentration of H_2_ and CO in the syngas decreases, resulting in a decrease in H_2_ production. The water–gas shift reaction and steam–methane reaction move toward the reactant side as pressure increases, causing the concentration of CH_4_ to slightly increase. Higher CO_2_ and CH_4_ concentrations are produced by an increase in pressure. The Boudouard reaction is responsible for the change in CO_2_ concentration; thus, as it reverts at greater pressures, the generation of CO_2_ is promoted. However, elevated pressure promotes the reverse water–gas reaction, reverse steam–methane reforming and reverse Boudouard, resulting in a decline in the concentration of H_2_ and CO. (4) At all temperatures, pressures, and ERs, the gasification of PP generates the maximum H_2_, whereas the lowest H_2_ yield in the case of PVC. The yield of H_2_ produced by using five distinct polymers reached 285 Nm^3^ H_2_ per ton feed and depending on the characteristics of the plastics used. The highest H_2_ yield was produced at a temperature in the range of 700 °C and 1200 °C, ER in the range of 0.10 to 0.15, and a gasification pressure of 1 bar.^103^

In the case of fluidized bed reactors, particle agglomeration phenomena impair their large-scale operation, deteriorating the fluidization quality, and ultimately resulting in defluidization.^104^ Plastics become sticky when heated at high temperatures, and thus the “coating-induced” process leads to clustering in gas–solid contactors during the pyrolysis of plastic, where a sticky layer forms on the particle surface.^105^ The stickiness, velocity, and surface contact of the particles will determine their propensity to aggregate.^106^ Small cumulations of bed material often form first, and when these agglomerates grow, they may cause the bed to collapse. The plastic particles supplied to the reactor initially have a soft exterior; however, their center remains cool because of their weak thermal conductivity.^107^ The softer surface turns sticky and creates aggregates of plastic particles encircled by sand particles from the bed in the reactor. Coating nearby sand particles with the softened plastic particles results in their distribution; if the thickness of the coating layer exceeds a threshold point, the sand particles will fuse together.^108^ In this case, increasing in the ratio of sand and plastic in the bed is the only way to minimize the interaction. The fused thick plastic covers the sand, preventing defluidization in the fluidized bed. As a result, the process yield is reduced given that a lot of sand is needed to encourage fluidization, requiring reactors with greater volume, increased gas flow rates, and higher energy.

### 4.2 Conical spouted bed reactor

Generally, the use of a typical conical spouted bed reactor for PW pyrolysis is evaluated to give high performance based on its substantial rate of heat transfer and turbulence in its bed, leading to the minimization of particle aggregation challenges compared to fluidized bed reactors. Fig. 4(b) shows the strong solid circulation in a traditional conical spouted bed reactor, which permits high heat and mass transfer rates as well as isothermal operation with nearly perfect solid mixing.^109^ Moreover, fitting a fountain confiner in conical spouted bed reactors was studied to change the bed hydrodynamics, which extended the residence duration of volatiles and enhanced their interaction with the catalyst. Additionally, it was possible to work with finer materials, which improved the bed turbulence, gas–solid contact, and the *u*/*u*_ms_ ratio. Additionally, the confiner prevented fine elutriation and endowed the bed with excellent stability. The fountain confiner improved the cracking of tar, leading to efficient biomass gasification. As a result, it was evident the there was a decrease in tar concentration at 850 °C from 49.2 g Nm^−3^ to 34.6 g Nm^−3^ without the fountain confiner. Additionally, there was a notable improvement in the efficiency of carbon conversion and the generation of gas and H_2_. Additionally, it proved feasible to run under an enhanced fountain regime, which is characterized by high bed turbulence and considerable fountain, by reducing the size of the olivine bed particles. Because of these characteristics, the olivine and gases could make better contact, which reduced the syngas tar concentration to 20.6 g Nm^−3^, as highlighted in Fig. 4(c).^110^ A recent study investigated the selective production of H_2_ and the valorization of plastic waste using pyrolysis and in-line oxidative steam reforming. To guarantee a uniform O_2_ distribution and avoid catalyst deactivation, a multi-point O_2_ injection system was created. A conical spouted bed and fluidized bed reactor were used in a two-step system to test the pyrolysis and in-line oxidative steam reforming of high-density PE. At the optimal temperature of 700 °C, steam/plastic ratio of 3, 12.5 g_cat_ min g_HDPE_^−1^, and ER of 0.2, it yielded 25.0 wt% H_2_, which was 28.6% lower than traditional pyrolysis–steam reforming. However, in contrast to the traditional steam reforming process, the presence of O_2_ in the reforming reactor caused the pyrolysis volatiles to partially oxidize, which decreased the amount of H_2_ produced.^111^ Barbarias *et al.* studied high-density PE, which was flash pyrolyzed at 500 °C in a conical spouted bed reactor, and then steam–reformed in a fluidized bed reactor using a commercial Ni catalyst as part of a continuous process. Complete conversion was achieved at 700 °C, 16.7 g_cat_ min g_HDPE_^−1^, and a steam/plastic ratio of 5, where the H_2_ yield was 92.5% of the stoichiometry-corresponding yield, namely 38.1g_H_2__/100 g_plastic_.^112^

### 4.3 Rotary kiln reactors

**Fig. 5:**
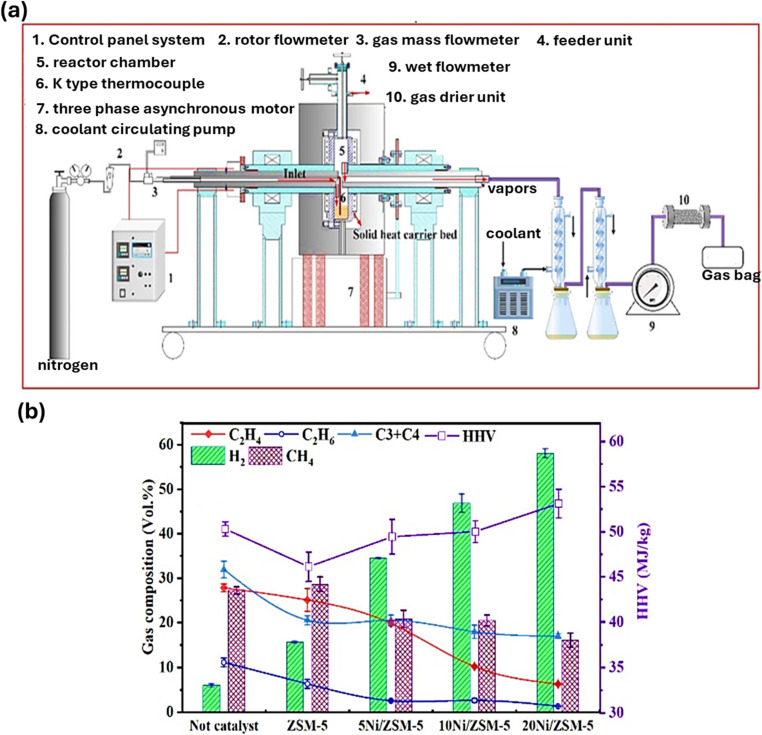
(a) Schematic overview of the semi-batch rotary reactor and (b) gas composition, specifically H_2_. Reproduced with permission from ref. 116. Copyright 2024, Elsevier.

Rotary kiln reactors are also employed for plastic pyrolysis, especially on an industrial scale because of their ability to handle irregular particles with different heat capacities. The heat input and residence time can be controlled by altering the speed of the screw, affecting the distribution of product. Rotating kiln reactors can control the mixing to maximize the product dispersion.^99^ Compared to fixed bed reactors, rotary kilns provide superior heat exchange to the feedstock and have easy operation compared to fluidized bed reactors. The residence time of the feedstock within the reactor is a significant factor in pyrolysis given that it influences the energy that the charge receives at a particular heating rate.^113^ The residence duration in the rotary kiln reactor is frequently a function of the mean volumetric flow and the rotation rate of the kiln. To produce more uniform pyrolytic products, the moderate rotation of the inclined kiln allows good mixing.^114^ However, although the heating is consistent, it is comparatively given that heat is only transferred through the reactor wall. Conventional pyrolysis, also known as slow pyrolysis, is frequently performed in these reactors at 500 °C for a residence time of 1 h.^115^ Although they have a simple design and operation, these reactors are just as adaptable as the conical sprouted bed reactor for handling mixed plastic with different forms and sizes.^2^ Zhang *et al.* explored the *in situ* catalytic pyrolysis of polyethylene employing a semi-batch rotary kiln reactor (Fig. 5(a)) having solid carriers for heat and Ni/ZSM-5 catalyst for producing BTX (benzene, toluene, xylene) aromatics and H_2_. This study demonstrated that the transfer of heat in a rotary reactor can be improved by loading more solid heat carriers, and the materials inside the solid heat carrier bed can be heated to 200 °C per second. Increasing the thickness of the active layer and accelerating particle mobility in the solid heat carrier bed allowed for this improvement. Upon mixing a catalyst in the solid heat carrier bed, the catalyst particles and solid heat carriers will move, leading to the continuous and effective interaction of volatiles evolved from the pyrolysis. As shown in Fig. 5(b), H_2_ production increased to 58.0 vol% in the presence of a catalyst compared to without the catalyst, which was only 6 vol%. Also, it was found that a lower loading of catalyst in the *in situ* catalytic pyrolysis may lessen its contact with the solid heat carrier, which would affect the overall cracking capacity.^116^

### 4.4 Microwave-assisted reactors

**Fig. 6:**
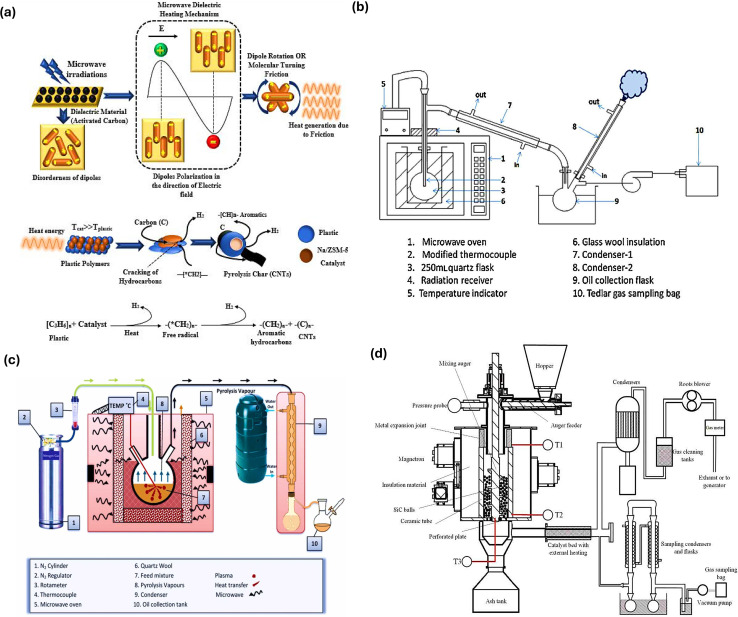
(a) Schematic overview of the mechanism involved in the contact of microwaves with a dielectric to carry plastic pyrolysis. Reproduced with permission from ref. 118, Copyright 2023, Springer. (b) *Ex situ* microwave pyrolysis reactor. Reproduced with permission from ref. 125. Copyright 2015, Elsevier. (c) *In situ* microwave pyrolysis reactor. Reproduced with permission from ref. 122. Copyright 2022, Elsevier. (d) Illustration of a continuous microwave-assisted pyrolysis reactor system. Reproduced with permission from ref. 126. Copyright 2021, Elsevier.

Other technological advances, including microwave-assisted reactors and plasma reactors, are still in the initial phases of advancement, in addition to other reactors. The design of the experimental apparatus influences the plastic distribution system in microwave pyrolysis reactors. Microwave ovens, containers for reaction, temperature sensors, gas condensers, liquid collecting containers, gas bags, and insulation materials are some of the vital experimental components. The tightness of the entire system and the matching of every part are essential for the effective design of the experimental device.^117^Fig. 6(a) illustrates the mechanism involved in the contact of microwaves with a dielectric to carry out plastic pyrolysis. As a dielectric substance, activated carbon interacts with charged particles in the material to absorb microwaves and produce heat. Dipole polarization, in which dipoles come in line with the oscillatory electric field, and dipole rotation, in which polar molecules continually reorient, lead to heat generation. Heat is produced by the friction caused by these movements. A study used a sodium zeolite catalyst, where the heat produced breaks down the longer-chain hydrocarbons selectively and speedily, producing shorter-chain hydrocarbons (aromatics or alkenes wax) as well as solid carbon residue and H_2_.^118^ Currently, most of the investigations on microwave-assisted waste plastic pyrolysis is done in batch-type reaction apparatus in laboratories. As depicted in Fig. 6(b), among the microwave pyrolysis reactors, *ex situ* reactors typically consist of a generator for generating microwaves, a closed lid to prevent radioactive leaks, and an exterior collection device for gathering produced liquids and gases separately. Mostly, these types of reactors have a capacity of <20 g per batch.^119–121^ In the case of an *in situ* microwave pyrolysis reactor (Fig. 6(c)), the container for the reaction is positioned into the middle of the reactor and is attached to a temperature sensor, an apparatus to condense steam, and a gas purge intake. For instance, in the current work, a borosilicate vessel was utilized. Firstly, a microwave oven was employed for direct pyrolysis, while the second one served as a source of heat for the catalytic reforming of volatiles. *In situ* microwave pyrolysis slows down the deactivation of the catalyst to some extent due to carbon deposition and is more favorable for the regeneration and separation of the catalyst from the reactants.^122^ Aishwarya *et al.* engineered a batch microwave reactor using a quartz reactor, condensers, a cold trap, and a microwave oven operating at 2.45 GHz and having a shifting output energy of up to 5 kW, making it suitable as an industrial-scale microwave reactor. The ideal pyrolysis process resulted in the creation of a product fit for use as fuel. Moreover, the reactor showed the advantage of using different types of impure plastics instead of one type, whereas the absorbent for the microwave was carbon.^123^ Recently, a system for continuous microwave-assisted pyrolysis was created (Fig. 6(d)). With an overall microwave output capacity of 9 kW, it included a downdraft mixed bed made of silicon carbide. An auger feeder having a 10 kg per hour capacity could constantly input the feedstock, while it was placed in the hopper. Thus, the continuous reaction system is one of the most promising research avenues for the industrialization of microwave-assisted pyrolysis.^124^

**Fig. 7:**
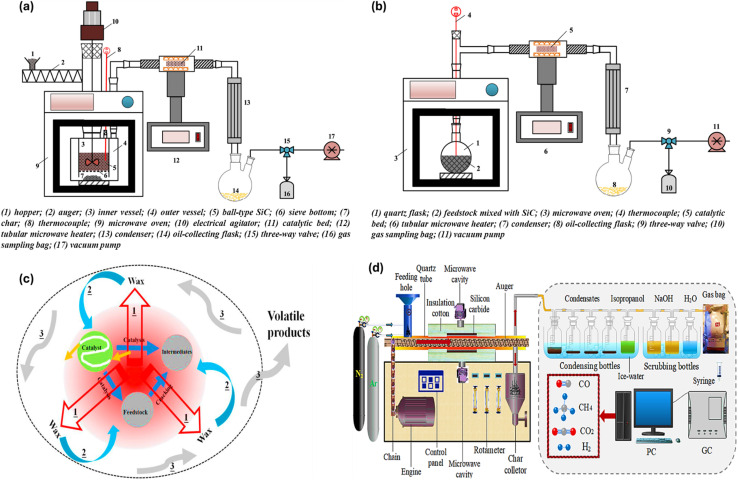
(a) Illustration of a continuously stirred and (b) batch phase microwave pyrolysis reactor. Reproduced with permission from ref. 127. Copyright 2021, Elsevier. (c) Overview of the contact between an absorber, plastic feed, and catalyst in microwave-assisted pyrolysis reaction. Reproduced with permission from ref. 128. Copyright 2021, Elsevier. (d) Experimental setup of screw rod-driven auger microwave pyrolysis continuous reactor. Reproduced with permission from ref. 133. Copyright 2022, Elsevier.

Fan *et al.* conducted a related study on a reactor for continuous-stirred microwave pyrolysis aided by a stirring unit and batch pyrolysis setup operating *via* microwaves to convert linear low-density polyethylene into fuels under similar conditions (Fig. 7(a and b)). In the case of gas products with a higher percentage of CH_4_ and short carbon chains, *i.e.*, C_7_–C_11_, the batch approach was more selective. The continuous-stirred system produced higher condensed products and was selective towards long carbon chains, *i.e.*, C_14_–C_20_, due to the increase in rotation, which led to uniform temperature and inhibited the excessive heating of long-chain compounds and their breaking to non-condensable smaller compounds because of the hotspot effect. Similar product yields were seen for both configurations in the comparison of catalytic processes, with the main difference being in the chemical species selectivity.^127^ Moreover, a study further explored the influence of the parameters and mode of mixing and heating, ratio of the absorber, and the volume of the pyrolysis reactor on the end-product using high-density PE with activated carbon as an absorber and molecular sieve as the catalyst. The temperature distribution was greater in the center compared to the edges due to the internal and volumetric heating and contact of the absorber with microwaves, leading to the fast and direct discharge of product, as shown by Paths 1 and 3 in Fig. 7(c). Wax production can be encouraged by using continuous heating, shortening the residence period, and using less activated carbon, and a high yield of 87.75 wt% was reported. Increasing the residence duration and activating carbon, while using intermittent heating and mixing aided the generation of liquid products (C_7_–C_20_), with the highest production being 82.36%. As shown in Fig. 6(c), in the case of Path-2, the products return to the absorber to further undergo a pyrolysis reaction, getting higher chances to interact with heat and the catalyst present.^128^ As shown in Fig. 7(d), a screw rod-driven auger microwave pyrolysis continuous reactor was designed, where there was a horizontal cylindrical reaction vessel that forced the plastic feed over a screw rod, achieving an elevated rate of recovery for organic materials from the plastic feed, *i.e.*, circuit boards. In another work, a continuous downdraft microwave-assisted pyrolysis system was reported, having 10 kg per hour capacity for plastic feed input. The reactor consisted of an auger feeder for feed having an auger shaft and silicon carbide balls. The auger shaft assisted in continuous mixing, leading to heat transfer and mass transfer with microwave radiation heating the ball bed. This makes the entire setup applicable on a larger scale, given that it reduced the processing temperature and showed higher efficiency than traditional fluidized bed reactors.^126^

In the context of H_2_ production, microwave-assisted pyrolysis is a simple and quick process for the catalytic breakdown of several plastic feeds into H_2_ and high-value carbons. For the initiation of catalytic breakdown, microwaves, in conjunction with cheap and widely available iron-based catalysts, acting as microwave susceptors were employed. A commercial plastic sample that was mechanically ground was converted into H_2_ and multiwalled C nanotubes in a single phase, which took 30 to 90 s. H_2_ generation of 55.6 mmol was accomplished, extracting more than 97% of the potential mass of H_2_ from the disassembled plastic.^49^ In another study, a novel method of using microwave-induced reactions over Fe/Ni–CeO_2_@CNTs to produce 91.5 vol% H_2_ using non-recyclable plastic waste was reported. The effective breakdown of C–H bonds, which was supported by Fe/Ni components energized by electrical discharge and microwave-induced “hot spot” effects, led to the maximum H_2_ generation within seconds. The contact between the relatively cool interior of the plastic and the active sites was the location of the reaction. The H_2_ concentration remained over 85% during the process, which produced H_2_, CH_4_, and C_2+_ hydrocarbons. As Fe/Ni oxides were reduced over time, the amount of CH_4_ increased and CO decreased. After the carbon deposit was oxidized to CO, it reformed into H_2_ and CO. Close to the completion of the reaction, thermal cracking was dominant, increasing the production of CH_4_. This highlighted the role of microwave irradiation in promoting significant H_2_ generation by catalyst activation and strengthening of bond cleavage.^129^ Shen *et al.* explored microwave-assisted pyrolysis for the thermal treatment of high-density PE for conversion into H_2_ and CNTs with the help of iron-based catalysts using microwave-encouraged ‘micro-hot spots’ theory. The yield of products and the compositional nature are dependent on the efficiency of the catalysts used as well as their ability to absorb microwaves. High-density PE was fully pyrolyzed following 1-h microwave irradiation at 500 °C. Among the non-condensable gaseous products, about 96.8% was H_2_ and 3.20% was CH_4_.^130^ Another study examined a 10% Fe/Al_2_O_3_ catalyst for the production of more than 92% H_2_ by microwave-mediated single-step pyrolysis, together with the synthesis of a useful carbon nanotube. Therefore, it has been demonstrated that microwave pyrolysis is a more practical and efficient method than traditional heating.^131^ Li *et al.* studied microwave radiative thermal processing using Al–Fe catalysts for the conversion of PP plastic waste to H_2_ and achieved an efficiency of 97.65% together with bamboo-shaped C nanotubes. Moreover, according to the variations in product distribution mechanisms, microwave assistance led to a four-fold increase in the yield of H_2_. Also, the discrepancy was compared using Monte Carlo risk analysis and the techno-economic assessment. Within 2.5 years, microwave technology generated $577 per tons of plastic with an internal rate of return of 39%.^132^

### 4.5 Plasma reactors

**Fig. 8:**
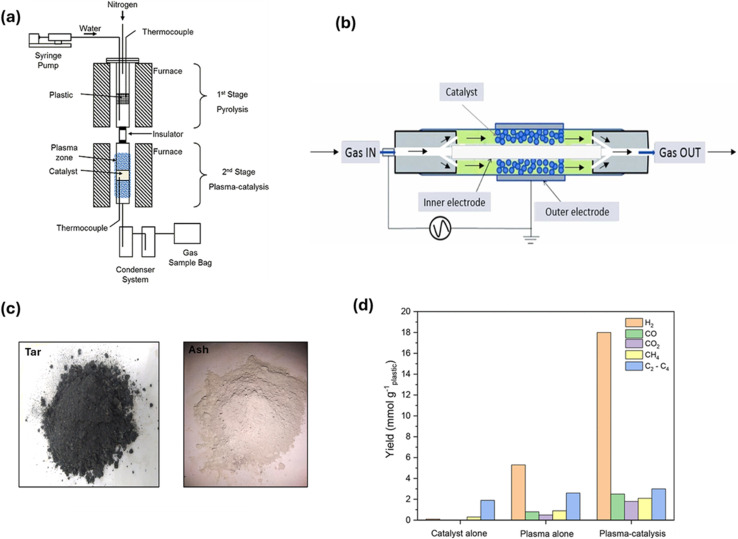
(a) Two-stage reactor consisting of pyrolysis and a DBD non-thermal plasma reactor. Reproduced with permission from ref. 136. Copyright 2022, the American Chemical Society. (b) Illustration depicting the flow of gases in a DBD non-thermal plasma reactor. Reproduced with permission from ref. 134. Copyright 2023, Elsevier. (c) Solid byproduct from the plasma pyrolysis reactor. Reproduced with permission from ref. 137. Copyright 2024, Elsevier. (d) Comparative analysis of H_2_ production and total gas yield from plasma under different conditions. Reproduced with permission from ref. 136. Copyright 2022, the American Chemical Society.

In a recent work, a two-stage reactor comprised of a pyrolysis reactor for the release of hydrocarbons and a dielectric barrier discharge plasma (DBD) non-thermal plasma reactor for steam reforming was explored for H_2_ generation.^134^Fig. 8(a) illustrates the reactor setup,^135,136^ where a DBD reactor and a stainless-steel pyrolysis reactor make up the two-stage reactor system. A container made of stainless-steel containing plastic was heated in a furnace from 20–500 °C. Then, the plastic was held at 500 °C for 15 min for it to undergo pyrolysis. In the second step, the catalyst was positioned in the DBD plasma reactor discharge gap, stabilized with quartz wool, and kept at 250 °C to avoid steam and pyrolysis hydrocarbon condensation. The catalyst was inserted in the DBD plasma reactor, which was made of a quartz tube having an inner aluminum rod as well as an exterior copper electrode. The catalyst was kept between the electrodes. Steam was added using a syringe pump. Then, the gases generated were collected using a condenser system to gather liquids and placed in a gas sampling bag. Fig. 8(b) illustrates the flow of gases within the reactor involving gases from pyrolysis and N_2_, used for purging. The generation of plasma takes place when the electric current is passed between the electrodes present outside and inside, leading to electric discharge due to the significant difference in potential, causing gas ionization and generating plasma. The product yield and product distribution were guided by the structure and composition of the plastic polymer, as evidenced by the subsequent breaking of pyrolysis volatiles from different polymers utilizing pyrolysis plasma catalysis with no steam. The C–C bond and C–H bond were broken at lower temperatures by electron impact reactions, which caused cracking to occur. The C–CH_3_ bond needs lower energy for bond breakage compared to the C–H bond, which leads polypropylene generating more gas compared to other polyolefin polymers. When steam was added to the system to reform the hydrocarbons produced by pyrolysis, it was demonstrated that steam reforming took place at a relatively low experimental temperature, producing CO and H_2_. Compared to plasma breaking (without steam), the H_2_ yield was higher.^134^ In another work, a plasma pyrolysis reactor was used to treat different types of plastic waste at 700–1000 °C and 2.5–10 kg per hour flow rate. The plasma pyrolysis technique is advantageous given that it has been shown that increasing the temperature increases the amount of H_2_ produced, while decreasing the amount of solid residue. In a continuous reactor, it is simple to reach high temperatures and a higher rate of heat transmission. As shown in Fig. 8(c), only tar and ash were produced as solid by-products in small amounts at high temperatures, showing that the production of solid products was minimized. The endothermic breakdown of polymeric chains is energy-intensive, but this approach appears to be clean because the amount of CO_2_ and tar produced was small in comparison to alternative techniques. Because the thermal plasma process was comparatively quicker, it was appropriate for continuous processing and resulted in a smaller reactor volume. The amount of waste that was handled using this technology was greatly reduced.^137^ Aminu *et al.* investigated the use of two-stage pyrolysis nonthermal plasma/catalytic steam reforming reactor for H_2_ production using Al_2_O_3_, TiO_2_, dolomite, BaTiO_3_, CaTiO_3_, Mo_2_C, Y-zeolite, ZSM-5, and MCM-41 as catalyst support materials. Although some of the materials improved the formation of surface discharge and micro-discharge, others hindered the generation of plasma. The maximum yield of H_2_ was generated by MCM-41, *i.e.*, 11 mmol g_plastic_^−1^. The catalyst and plasma environment worked together to produce a synergistic effect, which boosted the generation of H_2_ as well as the yield of total gas compared to total gas production using only catalyst or only plasma without a catalyst (Fig. 8(d)). The size of particles and the depth of the catalyst bed influenced the total gas rate and plasma discharge. Due to the improved surface reactions, impregnating nickel onto MCM-41 working as a support promoted H_2_ production, *i.e.*, 18 mmol g_plastic_^−1^.^136^

**Fig. 9:**
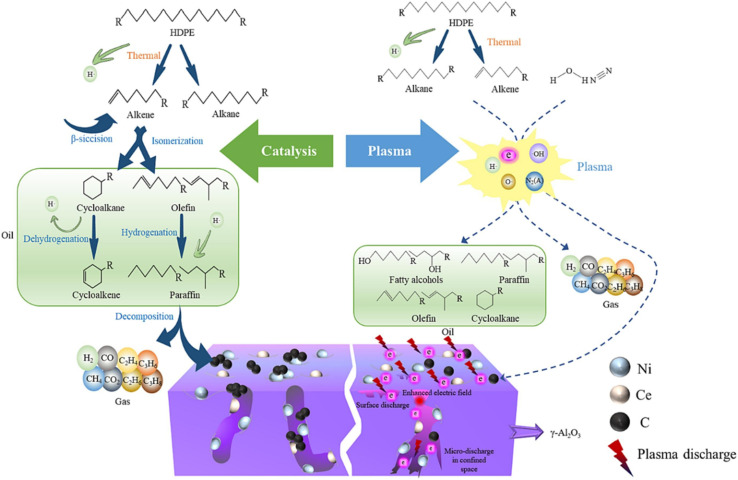
Overview of the reaction mechanism for the pyrolysis of high-density polyethylene and plasma–catalysis using the Ni–Ce/γ-Al_2_O_3_ catalyst. Reproduced with permission from ref. 138. Copyright 2024, Elsevier.

Ma *et al.* explored the effective plastic-to-H_2_ conversion of high-density PE using a combined pyrolysis and plasma–catalysis reforming setup. They reported that the strong synergy between the catalyst and plasma resulted in the evolution of H_2_ at different reforming temperatures, while a synergistic effect of 250.98% at 500 °C was reported. The plasma–catalysis reforming produced a total gas yield of 146.50 mmol g^−1^ and H_2_ yield of 102.52 mmol g^−1^, which were three-folds higher than the gas yields from the catalysis and plasma-alone reforming alone. As shown in Fig. 9, a sequence of processes, including β-scission, isomerization, hydrogeneration, and others, broke down heavy hydrocarbons into light hydrocarbons when catalysis was used alone. However, the catalyst pores became blocked as a result of carbon deposition caused by the reactions. Regarding the plasma–catalysis system, the plasma increased the catalytic performance and intensified the reaction by generating electrons with high energy and different free radicals in the plasma electric field, which encouraged pre-cracking of the volatiles obtained from pyrolysis. The plasma also reversed defects and acid sites created on the surface of the catalyst, which the maximized the catalytic performance and reaction efficiency.^138^

**Fig. 10:**
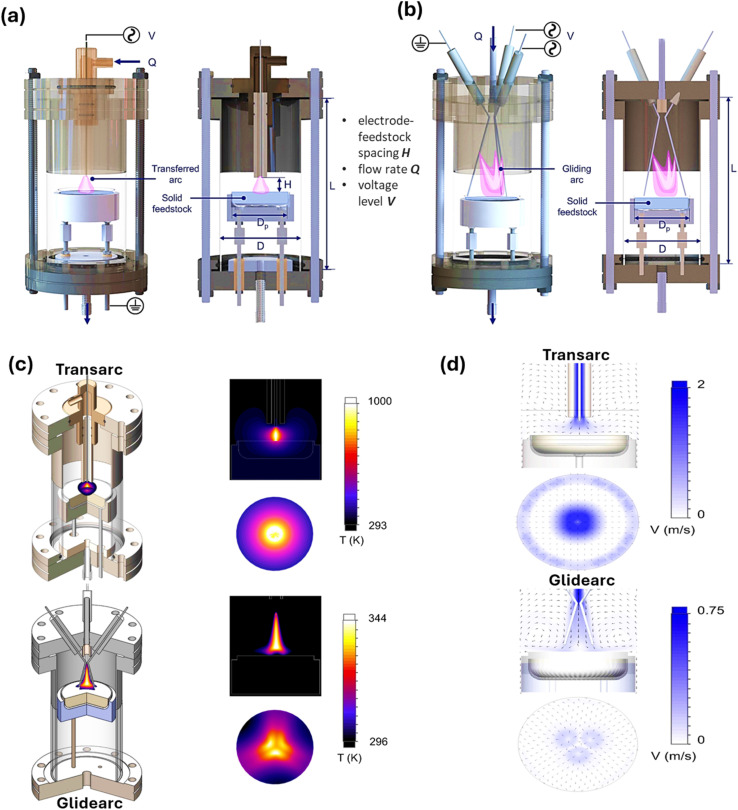
Structural overview of (a) the transarc and (b) glidarc low-temperature atmospheric pressure plasma reactors, (c) computational thermal fluid models for transarc and glidarc reactors, along with the temperature distributions and (d) velocity distribution for the transarc and glidarc reactors. Reproduced with permission from ref. 139. Copyright 2022, Elsevier.

In a recent study, to generate H_2_ from low-density polyethylene, low-temperature atmospheric pressure plasma reactors were developed and investigated. As depicted in Fig. 10(a and b), the reactors were built based on transferred arc (transarc) electric discharge and gliding arc (glidarc) electric discharge. In the case of the transarc reactor, the distance that lies in the electrode tip and the feedstock served as a control parameter, which transfers electric current to the feedstock by means of a tungsten electrode situated above an aluminum disc. Alternatively, three tungsten electrodes, two powered and one grounded, were placed in a triangular pattern on the glidarc reactor. Gas influx and plasma buoyancy caused the arc to move smoothly along the electrodes. As can be clearly seen in Fig. 10(c), the temperature of the transarc was highest near the plasma center (about 750 K), and it decreased radially. The high thermal power/unit volume was caused by the limited plasma volume. The temperature distribution of the glidarc exhibited a three-fold symmetry, with the greatest feedstock temperature being close to 300 K, suggesting non-uniform heating. In contrast to the transarc, the simulation forecasted a wider plasma interaction area with the feedstock. The reactor walls stayed around room temperature (300 K) despite the high plasma temperatures, indicating that the reactor can function close to room temperature without further cooling. Moreover, in both reactors, vortex rings were observed to be formed close to the surface of the sample in the flow field (Fig. 10(d)). The glidarc reactor showed more uniform treatment because of its lower velocity, and the transarc reactor had greater velocity around the center, which resulted in the formation of a crater-like pattern. The comparative production of H_2_ from low-density PE was studied for both reactors. The turbulence and plasma residence time in the transarc and glidarc reactors, respectively, were the factors that affected H_2_ production. H_2_ production increased with an increase in the voltage in both reactors. Electrode-feedstock spacing was important in the transarc reactor, while flow rate was important in the glidarc reactor. However, both reactors had higher energy costs than traditional methods, and despite their operational differences, both reactors showed comparable H_2_ production.^139^

In the case of plastic pyrolysis, the catalytic systems are usually adapted to the reactor design to maximize their stability and performance. For instance, based on their high acidity and structural fragility, zeolite-based catalysts (such as H-ZSM-5 and ultra-stable Y zeolite) are frequently employed in fixed-bed reactors. At the same time, their applicability is restricted in high-attrition settings.^164^ Alternatively, mechanically robust catalysts that can tolerate continuous movement, thermal cycling, and *in situ* regeneration, such as metal–supported oxides (Ni/AlO_3_) and spent fluidized catalytic cracking catalysts, are frequently used in fluidized bed reactors.^165,166^ Moreover, attrition cannot stop catalyst deactivation, regardless of the catalyst utilized in the process; hence, a catalyst regeneration strategy is needed.^167^ In a study, a continuous process comprising the flash pyrolysis of high-density PE in a conical spouted bed reactor and catalytic steam reforming having volatiles produced in a reactor with a fluidized bed was used to examine the performance of an Ni catalyst in the reaction–regeneration cycles. A range of air concentrations and temperatures of 600 °C to 700 °C was used for *in situ* coke combustion in the reforming reactor to regenerate the catalyst between operations. However, due to the elevated temperatures required for its regeneration, sintering of the Ni^0^ active sites occurred, diminishing its catalytic ability.^157^ In a study, a mixture of H-ZSM-5 and Al-MCM-41 catalysts in a fluidized bed reactor was explored for catalytic pyrolysis. This study carried out multiple regenerations of the catalyst after subsequent runs, indicating a decline in its ability, but the surface area was regained up to 94%.^168^ Thus, proper regeneration strategies can help to regain the activity of catalysts to some extent.

## 5 Production of value-added fuels *via* catalytic pyrolysis

When PW is pyrolyzed, three main products are usually produced, carbon-rich solid char, non-condensable gases such as C_1_ to C_4_ from polyolefins, and CO, HCl, and CO_2_ from polycarbonate (PC) and PVC, as well as plastic oils, which can be liquid or waxy. In the case of fuel oils, their yield is in the range of 80% to 90 wt%, which can be retrieved from thermoplastics.^115^ According to Zhang *et al.*, a simulated mixture of plastic was studied to give an exergy efficiency of 60.9% to 67.3% and an energy efficiency of 59.4% to 66.0% in a pyrolytic rotary kiln reactor.^169^ However, to increase the efficiency of this process, HydroPRS technology works on plastic pyrolysis but without producing char or residue, increasing the efficiency upto 80% to 90%.^170^ Long-chain alkanes and alkenes larger than C_20_ with high boiling temperatures (>500 °C) make up waxy plastic oils. These compounds must be further broken down, for example, by fluid catalytic cracking, to produce liquid fuels or other petrochemical commodities.^155^ Conversely, aliphatic chemicals and mono-and polyaromatics make up the majority of liquid plastic oils, which can utilized as obtained for the generation of power in steam boilers, as fuel in transport, and for the generation of C nanotubes.^171^ Also, char can be used to prepare adsorbents, AC, graphene and its derivatives, and the non-condensable gases are utilized again as the heat source in pyrolysis.^172^ The efficiency of the primary product is dependent on the residence time, temperature, and heating rate,^3^ whereas the product distribution is dependent on the type of PW, type of reactor, heating mechanism, pressure, and catalyst employed.^173^

### 5.1 Liquid plastic oils

Recently, Aisien *et al.* employed thermal and catalytic slow pyrolysis and explored the conversion of high-density PE PW to high-quality liquid oils at a low temperature of 500 °C using a 0.2 catalyst-to-plastic ratio. The products included 88.8%, 9.9%, and 1.3% liquid oil, gases, and char, respectively. In comparison to thermal pyrolysis, catalytic pyrolysis yielded less gases but more liquid oils (C_6_ to C_24_), including 10.5% motor oil, 39.5% kerosene, 50% diesel, and 47.7% gasoline.^174^ Another study employed a bentonite clay catalyst that was pelletized, generating C_5_–C_9_ hydrocarbons, *i.e.*, gasoline from PS, while PP, high- and low-density PE led to the generation of longer aliphatic hydrocarbons appropriate for use in diesel. To catalyze 1 kg of PW, a reaction time of 10 min was required, and there was no wax formation due to pelletized bentonite. The produced fuel was reported to release less CO and CO_2_ compared to traditional fuel.^175^ Likewise, bentonite clay was used in catalytic pyrolysis at 450 °C. The PS and PP PW generated zero wax in pyrolysis oils. The production of gases increased, while the production of oils decreased in the presence of a catalyst in the case of PS, PP, and low-density PE plastics. In the presence of catalysts, PS was capable of producing the highest fraction of diesel. PS and PVC were found to be unsuitable for oil production due to the challenges of char, lower output, and increased energy needs.^176^ Catalytic pyrolysis was carried out using different municipal PW and an Ni–Mo–W/zeolite-based catalyst (Z-503), which aided the production of oils and lighter gases compared to thermal pyrolysis, where diesel (C_12_–C_24_) was the prominent product; however, thermal pyrolysis showed greater selectivity for oils.^177^ According to a few studies, the use of 100% plastic oil as a fuel leads to challenges such as knocking, noise, and less desirable combustion; thus, blending plastic oil with 20 wt% diesel improved engine functioning with regard to thermal potential as well as emissions.^178^ In another work, an up-graded zeolite Y catalyst with loaded IL-53 (Cu) was used for the production of liquid fuels, where the optimum parameters for useful fuel production were explored using response surface methodology, through experimental design. The temperatures to obtain gasoline (41.4%), jet fuel (73.1%), and diesel (36.1%) were determined to be 381 °C, 525 °C, and 523 °C, respectively. A catalyst loading of 2.5% for gasoline and jet fuel and 10.5% for diesel was also identified.^179^

### 5.2 Pyrolysis fuel gases

10–90% of the plastic feed is made up of non-condensable pyrolysis gas from plastics, and its release depends on the operating parameters and process design. Gas production occurs at high temperatures and requires a longer residence time than pyrolysis oils.^180^ Most PW generates hydrocarbon gases, mainly methane, ethylene, and butadiene. However, some other gases are also generated in small amounts, such as propane, butane, propene, and *n*-butane.^181^ To generate heat for the process, these gases are usually burned on an industrial scale. Moreover, non-condensable pyrolysis gas from plastics can replace natural gas as fuel due to its high calorific value. Alkanes, alkenes, dienes, and alkynes are mixed in plastic pyrolysis gas, in contrast to natural gas. Unsaturated hydrocarbons must be eliminated for use in gas engines, gas turbines, and fuel cells. Moreover, alkenes are the main constituent of pyrolysis gas and can be utilized for the synthesis of chemicals as a precursor, after the successful removal of alkynes.^182^ Some PW, including those from PVC, PC, and PET, are responsible for producing hydrocarbon gases as well as a few toxic gases (HCl, CO, and CO_2_), leading to undesirable effects on the environment and causing corrosion to metals.^181^ An increased calorific value of 42 MJ kg^−1^ and 50 MJ kg^−1^ exists for the gases produced from PP and PE PW, respectively, which can be used as the source of heat in industrial settings. Also, ethylene and propene are capable of being used in the manufacturing of chemicals as feed after proper separation from the gas mixture. Their use is also prominent for electric power generation and to fire boilers without any treatment.^183–185^

### 5.3 Pyrolytic char-derived fuel

Given that plastics are made from carbon-based molecules, their catalytic pyrolysis leads to the formation of char, which is considered a residual and has a high carbon content. Higher char formation takes place under slow heating and low temperature, as well as longer residence time.^115^ Char is known to be utilized as a fuel for combustion and gasification, and can be burned in cooking stoves in the form of a briquette. A study by Jamradloedluk *et al.*, reported the formation of char from high-density PE having a calorific value of 4500 cal g^−1^, and 1 kg of char in the form of a briquette was employed as fuel for boiling water.^186^ However, some chars produced specifically from PET, PVC, and PS have inorganics in them originating from additives and contaminants, leading to a lower calorific value and making them unsuitable as fuel.^115^

## 6 Circular economy aspect of catalyst-assisted pyrolysis

The circular economy concept involves the “closing the loop” theory for the lifetime of materials/products and the entire life cycle of materials, ranging from their generation, use, and then their discard and management of waste to the recovered resources market.^187^ The manufacturing process known as “closed-loop recycling” closes the material and energy loops by reusing and recycling post-consumer goods to create the raw materials and energy required to create new iterations of the same product.^188^ This approach complies with the principles of a circular economy by turning waste into valuable resources and promoting the recycling and resource efficiency.^189^ Pederson *et al.* stated that high-density plastic trash can be used to create a variety of useful items.^190^ Moreover, high-density PE can be pyrolyzed to create oil, which can be combined with ethanol and ethoxy-ethyl acetate to replace diesel in industrial and marine engines. This gasoline blend is more ecologically friendly, more efficient, and releases fewer emissions.^191^ In this case, compared to conventional pyrolysis, which requires inert gases to avoid side reactions, vacuum technology has been demonstrated to reduce the temperature and energy needs.^192^ Additionally, pyrolysis of biochar and PP copolymer to produce C nanotubes provides a sustainable method for turning waste plastics into useful resources. Furthermore, bitumen and wax from plastic pyrolysis can be used to make environmentally friendly paving materials, offering a circular solution in the paving business.^193^ Closing the loop of ethylene monomer production may be made easier by pyrolyzing PE to create ethylene. With a competitive pricing of 0.386 € per kg compared to 0.835 € per kg for the conventional naphtha-based technique, the results showed that recovered ethylene is a win–win solution.^194^ Moreover, gases from PP copolymer slow pyrolysis can be converted into bamboo-type CNTs.^195^ Waste plastic can be used as an alternative feed material in the synthesis of CNTs *via* a two-step pyrolysis procedure involving the recovery of products and synthesis of CNTs using the catalytic vapor deposition (CVD) approach involving catalytic pyrolysis gases.^196^

A study on microwave-assisted heating *via* two-step approach to recycle waste plastic packaging, converting it into valuable products, reported the production of rich fuel gases, including H_2_, CH_4_, C_2_H_4_, and C_3_H_6_. The solid products, primarily CaO, TiO_2_, and SiO_2,_ were declared appropriate as absorbers for the subsequent cracking process due to their quick increase in temperature (30–115 °C min^−1^) in a microwave field. The spherical activated carbon used as an absorber in initial cracking showed a long lifespan. After one or two uses, its heating characteristic diminished, but after numerous uses, it remained relatively unchanged, which is crucial for future industrial applications. The liquid products, ranging from C_7_ to C_26_, obtained from the 2nd phase of the cracking process, were declared to be appropriate as diesel and gasoline fractions.^197^ In another work, pyrolysis with catalytic reforming was employed, and the results showed that 80 wt% of the liquid products had features of diesel with a 41.558 MJ kg^−1^ energy content. The generated solid byproduct, *i.e.*, char, can be utilized after activation for the treatment of wastewater, elimination of heavy metals, and getting rid of smell and smoke. Energy carrier gases such as H_2_, CO, and CO_2_ were also generated.^198^ Thermal plasma technology is also considered a clean technological advancement, given that much less CO_2_ and tar are generated in comparison to other approaches.^137^ Also, the generated char can be added to epoxy composites,^199^ used as an adsorbent,^200^ and used in construction and electronics production, which is beneficial in advancing towards a circular economy.

The high heating values of the pyrolysis gas and oil, roughly 30 and 40 MJ kg^−1^, respectively, promote their direct use as fuel, whereas ordinary diesel, heavy fuel oil, and gasoline have also been reported to have high heating values.^201^ However, char is composed of a much greater carbon content that can also be used as a solid fuel or precursor (with a heating value of about 30 MJ kg^−1^).^202^ Thus, the production of value-added products *via* the pyrolysis of PW is advantageous to the overall economic performance of the waste system by enabling greater non-renewable resource savings, lowering PW disposal in landfills, releasing valuable products on the market instead of low-quality materials, and reducing carbon emissions.^188,203^

Among the policies directed towards curbing plastic pollution in the circular economy aspect, in 2023, the United States state-level advanced recycling legislation became the focus, which incorporated the chemical recycling of plastics. A total of 38 bills in 23 states was introduced and 8 were enacted. However, these laws are generally considered under manufacturing instead of PW management, leading to fewer regulatory obligations, but at the same time, some states have proposed the ban of these facilities, such as in Massachusetts and Rhode Island, due to concerns related to pollution.^204^ The U.S. Plastics Pact's Roadmap 2.0 focus on making the plastic industry more sustainable by focusing on advances for reusing plastic, minimizing single-use plastics, and circular design.^205^ The European Union's (EU), European Strategy for Plastics is involved in the circular economy package of the EU, focusing on reusable plastics, less costly recycling, and minimized use of microplastics.^206^ South Korea's Ministry of Environment waste policy is targeting Korea's transformation into a sustainable circular economy. In 2022, 110 national tasks were developed, among which the pyrolysis of PW into petrochemicals was one of them, focusing on the circular economy. This policy aims to improve the pyrolysis of PW by 10% by 2026. Also, it covers the provision of good-grade raw materials, expands the type of recycling as well as pyrolysis plants, and gives incentives for encouraging the pyrolysis process.^207^ Globally, the majority of countries consider plastic for incineration instead of chemical recycling. Moreover, there is a lack of regulations that cover the establishment, functioning, and management of pyrolysis plants. Thus, it might be advantageous for other nations to examine Korea's strategy for defining and controlling pyrolysis as a recycling step. A uniform approach for assessing and validating the contribution of chemical recycling is also required to achieve a global circular economy.^207,208^

Many companies are working towards the circular economy concept by recycling PW into valuables. Plastic Energy, Spain, was the first to commercialize plastic pyrolysis. They have two commercial pyrolysis facilities capable of handling 30 tons of end-of-life plastics. The facilities handle the processing of plastics and the pyrolysis of 5000 tons per year of mixed PW from households, 10% contaminated PW, and layered PW. The employed technology converts the PW into TACOIL (72–75%), which is 860 L per ton hydrocarbon oil (mainly diesel and naphtha) and is used by petrochemical industries. Also, 18% and 8% to 10% syngas and char, respectively, are also produced, where syngas is sold as a heat source, while char is utilized in construction works, contributing to a circular economy. Moreover, they plan to have 20 pyrolysis facilities in Europe and Asia, with a 500 000 ton capacity per year. In partnership with ExxonMobil, they plan to build a 25 000-ton plant in France. Saudi Basic Industries Corporation (SABIC) will be using TACOIL from Plastic Energy for manufacturing different types of plastics (light and high-density PE and PP).^209,210^ BioBTX uses Integrated Cascading Catalytic Pyrolysis (ICCP) technology for cracking plastics into aromatics. The mix of BTX is separated to get benzene, toluene, and xylenes with high purity, which can be employed for direct use as a substitute for fossil fuels, making them drop-in chemicals.^211^ The Pryme company uses mature pyrolysis technology to process plastic waste under high temperature and controlled reactor conditions without contaminants in the end-product. They aim to achieve 100% recyclability of plastics by powering the process through renewable energy. In 2024, this company successfully supplied its first shipment of 36 metric tons of pyrolysis oil.^212^ The SABIC TRUCIRCLE™ initiative aims at the development of circular polymers from reused PW. In Germany, SABIC produces naphtha to be used in the production of polymers, contributing to the circular economy. Moreover, it also produces renewable PE, PP, and PC, and uses renewable butadiene for producing styrenic block copolymers.^210,213^

## 7 Conclusion and future perspectives

• In conclusion, PW can be considered a renewable resource for the production of useful fuels such as H_2_ together with other products, including pyrolysis oils, char and other fuel gases. The treatment of PW can range from thermal to hydrolysis, photolysis, and pyrolysis processes. Among them, the pyrolysis process is considered most convenient industrial process. Utilizing proper catalysts can improve the catalytic processes by reducing the cost and time and minimizing the undesirable side products such as olefins, oil, and liquid products.

• The appropriate catalyst and support can maximize the H_2_ production at the minimum operating temperature. In the future, research should focus on exploring the operational parameters of the pyrolysis process, optimizing the catalyst/support for better H_2_ production, investigating and designing new catalysts for better selectivity and efficiency, exploring different PW for practical utilization, studying the limitations and barriers of the operational temperatures, flow rates, and design of reactors to maximize the desired product and investigate the effect of the feedstock on the production of H_2_. Moreover, less work is carried out to understand the mechanism involved in catalytic pyrolysis, which needs adequate computational and experimental approaches.

• Several variables, including the feedstock composition, the intended output, and operating limitations, affect the reactor selection. Fluidized bed reactors are typically known to be extremely efficient based on their excellent heat transfer capabilities, consistent temperature distribution, and high reaction efficiency, all supporting increased H_2_ yields. Microwave and plasma reactors heat rapidly and are capable of the selective activation of feedstock molecules for increased H_2_ production. Optimizing the H_2_ output in these reactors also heavily depends on the catalytic enhancement, especially in reactors such as fixed bed and spouted bed reactors, where the type and distribution of the catalyst significantly influence the process as a whole.

• Given that fluidized bed and microwave reactors have the greatest potential for producing H_2_ on a large scale, future developments should enhance their scalability and integration. The development of sophisticated catalytic materials that can tolerate severe pyrolysis conditions, while retaining good H_2_ selectivity should also be prioritized. Furthermore, integrating several reactor types, such as fluidized beds with microwave or plasma systems, can capitalize their complementary advantages and increase the H_2_ yields. To make catalytic pyrolysis a viable technique for producing H_2_ from PW on an industrial scale, issues with reactor durability, feedstock unpredictability, and economic viability must be resolved.

• The optimization of the catalyst–reactor combination for the catalytic pyrolysis of PW increase the process sustainability and efficiency. Reactor design and catalyst development have advanced significantly, but a systematic investigation connecting the two has mostly gone unnoticed. To customize solutions for various feedstocks and reactor types, future research should concentrate on investigating particular catalyst–reactor pairs using sophisticated modeling approaches and *in situ* characterization methods. In addition to progressing the effectiveness of PW pyrolysis, this will aid in the creation of ecologically benign, economically viable, and scalable recycling systems.

## Author contribution

The manuscript was written with contributions from all the authors. All the authors have approved the final version of the manuscript.

## Conflicts of interest

The authors declare no competing financial interest.

## Data Availability

No primary research results, software or code has been included and no new data were generated or analysed as part of this review.
